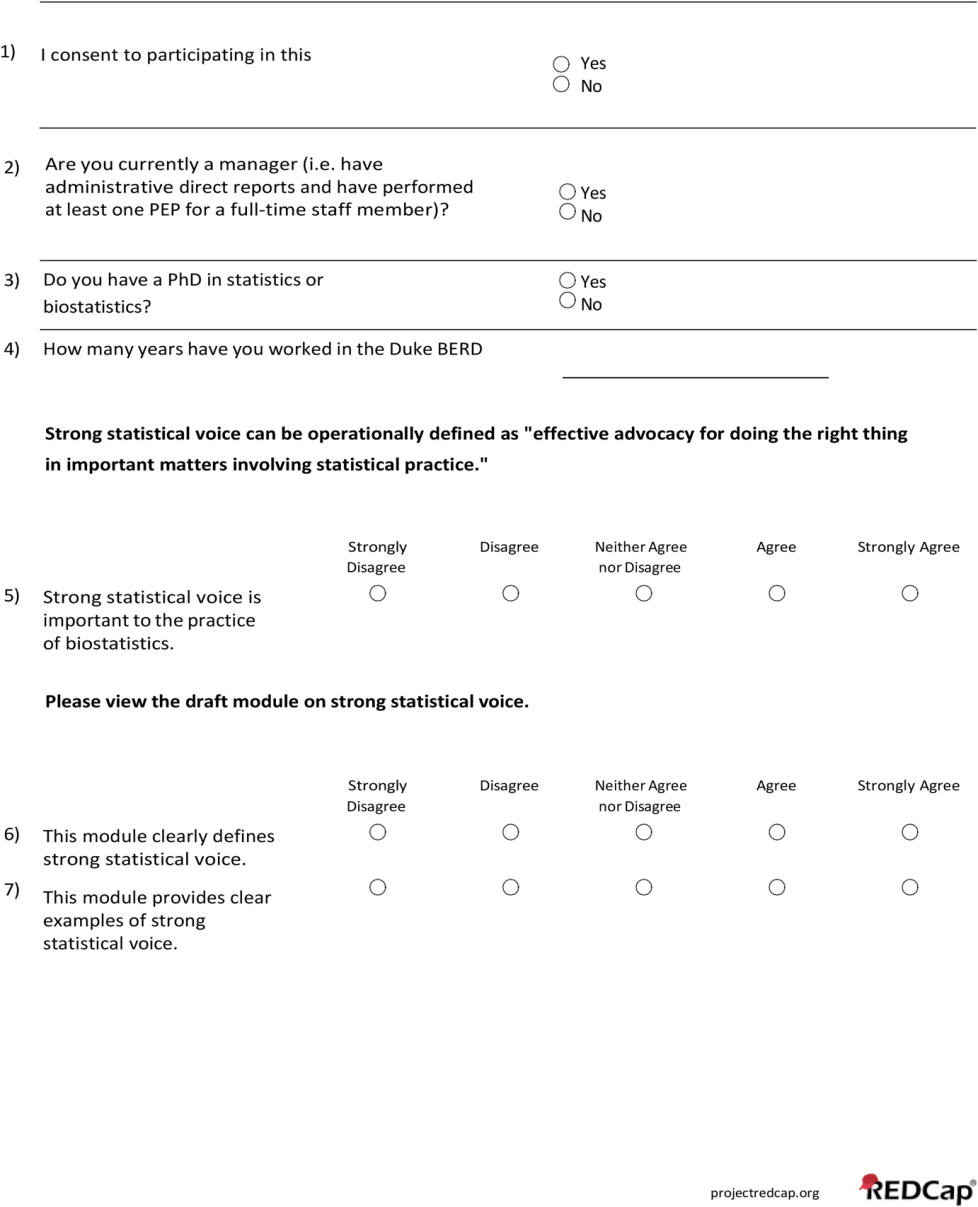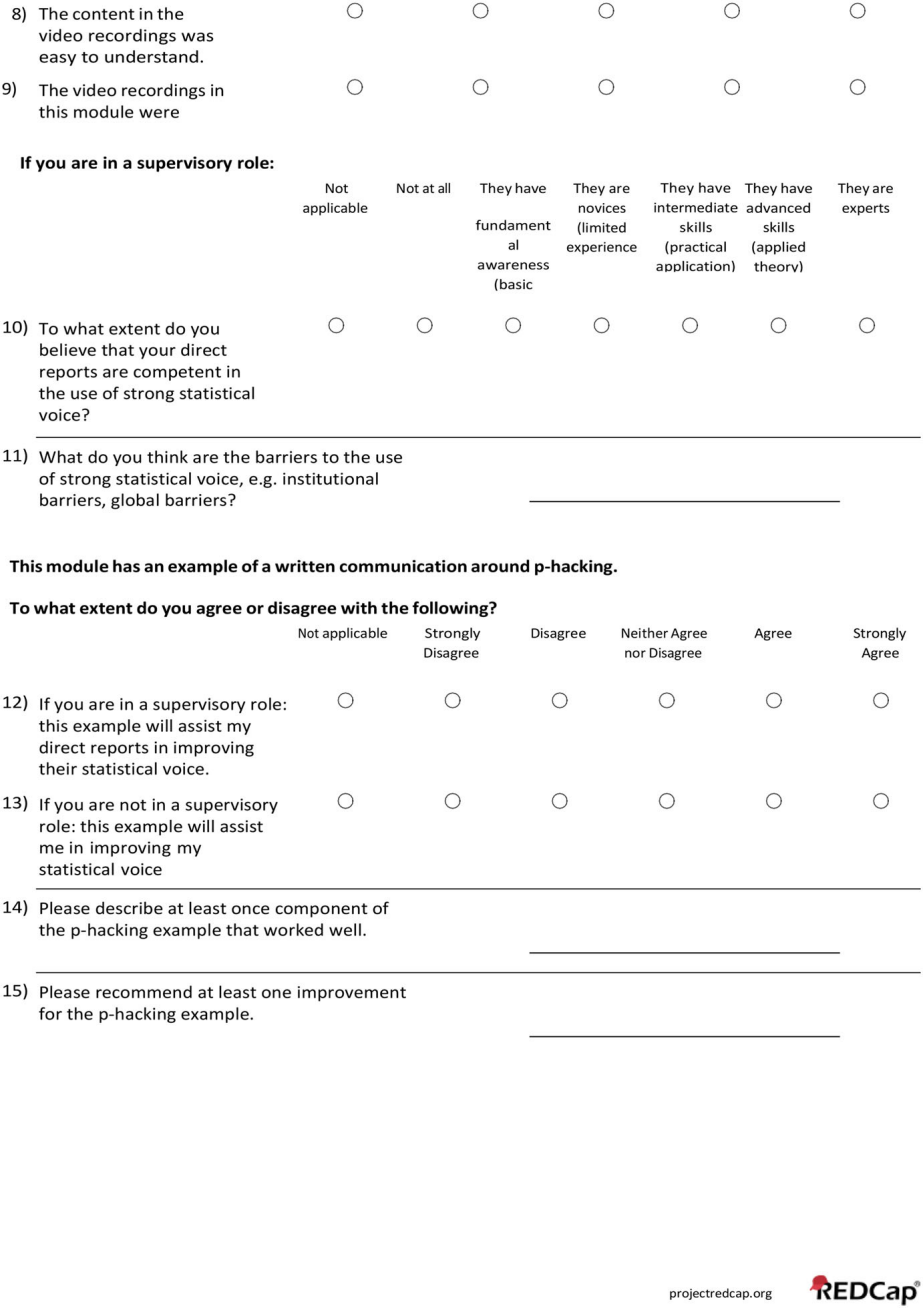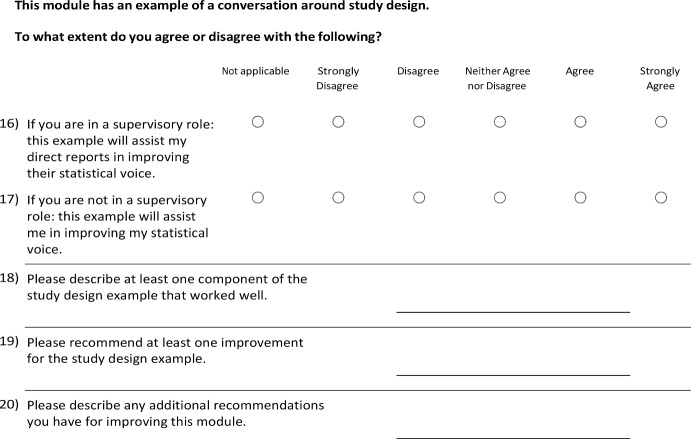# Enhancing Team Science by Training Collaborative Biostatisticians to have a Strong Statistical Voice

**DOI:** 10.1007/s42519-025-00522-7

**Published:** 2025-12-15

**Authors:** Gina-Maria Pomann, Steven C. Grambow, Marissa C. Ashner, Bibhas Chakraborty, Nan Liu, Megan L. Neely, Sarah Peskoe, Lacey Rende, Emily Slade, Tracy Truong, Lexie Zidanyue Yang, Greg P. Samsa, Jesse D. Troy

**Affiliations:** 1 Department of Biostatistics and Bioinformatics, Duke University School of Medicine, Durham, NC USA; 2 Centre for Quantitative Medicine, Duke-NUS Medical School, National University of Singapore, Singapore, Singapore; 3 Department of Biostatistics, University of Kentucky, Lexington, KY USA

**Keywords:** Professional development, Statistical competencies, Strong statistical voice, Team science training, Workforce development

## Abstract

Strong statistical voice is defined as the ability to advocate and negotiate for good and ethical statistical practices, including integrating and resolving differing scientific approaches. This skill is crucial for biostatisticians who work on biomedical research teams, as it ensures the integrity and accuracy of statistical analyses and fosters productive collaborations with non-statisticians. Despite its importance, new graduates often lack targeted training opportunities. This manuscript presents a scalable training approach through the development of online videos. Preliminary didactic materials focused on two key applications: providing written comments on manuscripts and engaging in study design discussions. To evaluate this training approach, a survey was conducted among biostatistics staff in the Duke Biostatistics, Epidemiology, and Research Design Core. The survey results indicated that all respondents strongly agreed on the importance of strong statistical voice in biostatistics practice. The clarity of the training materials and examples received positive feedback, though suggestions for improvement included enhancing video engagement and providing more hands-on training. This information will guide the development of formal training videos embedded within a mentored training program that aims to teach biostatisticians and other quantitative scientists how to effectively work on teams in biomedical research.

## Introduction

Quantitative scientists, including data scientists, biostatisticians, engineers, survey experts, and other scientists with specialized quantitative expertise, play a variety of important roles on biomedical research teams in academic healthcare centers [1–3]. They are often responsible for study design, data analysis, and appropriate dissemination of results within collaboration units in academic medical centers and across industry [4–8]. Collaborative biostatisticians work with research team members who have varied experience in data analysis and statistical methods. As a result, these collaborations require them to explain methodological choices, address conflicts about statistical approaches, and identify appropriate analytical compromises. Success in their roles requires mastery of multiple skills related to communication and leadership, developing quantitative expertise, and scientific domain knowledge [9–14]. Some skills are primarily technical, such as performing sample size calculations for various study designs. Others involve complex professional judgment, including the ability to apply ‘strong statistical voice’, which could also be described as confident quantitative communication. This refers to the ability to advocate and negotiate for good and ethical statistical practices, including integrating and resolving differing scientific approaches. [12, 13, 15]. As AI becomes more integrated into statistical work, the ability to think critically about analysis choices and clearly explain the reasoning behind them remains essential. Human judgment is key to guiding these tools and ensuring their outputs are valid, which makes strong statistical voice more important than ever in biomedical research [16].

Strong statistical voice was identified as one of 16 skills that collaborative biostatisticians need to master to contribute to biomedical research [12]. It was later identified through a national survey distributed to the workforce of collaborative biostatisticians as one of the top skills employers report as desirable and often absent in new graduates [13]. The survey results indicated a clear need for academic health centers and companies employing quantitative scientists to provide formal training in strong statistical voice. Until now, statisticians have primarily developed strong statistical voice through direct experience, observing colleagues, and individual mentoring. While effective for some individuals, these informal approaches lack the efficiency and scalability for systematic training. Sharp et al*.* developed online videos to train statisticians to improve their communication skills which do touch on some components of strong statistical voice, but there are not any targeted materials to teach this skill for quantitative scientists on biomedical research teams [19].

This manuscript presents a framework for training biostatisticians to navigate challenging conversations with non-quantitative team members and enhance their effectiveness within biomedical research teams. The framework includes online training videos with examples and activities evaluated on a sample of its target audience: collaborative biostatisticians embedded within multidisciplinary research teams at Duke University. Survey feedback from the Duke Biostatistics, Epidemiology, and Research Design Methods Core (BERD Core) is reviewed to assist in the development of scalable training resources. The feedback will inform current training within the unit and materials developed for the Quantitative Team Science Program funded by the National Institute of General Medical Sciences (R25GM155474). The manuscript first describes the target audience, next decomposes the critical components of strong statistical voice, then outlines methods for designing materials with tangible didactic value and concludes with considerations for future modifications and national program implementation.

## Developing Strong Statistical Voice: A Key Educational Objective

Collaborative biostatisticians work with research team members who have varied experience in data analysis and statistical methods. These collaborations require biostatisticians to develop and apply strong statistical voice, which involves explaining methodological choices, addressing conflicts about statistical approaches, and identifying appropriate analytical compromises. Strong statistical voice integrates technical expertise with communication skills, including articulating alternative analytical approaches and engaging in respectful yet direct dialogue about methodological decisions. Critical to this process is ethical reasoning, which guides biostatisticians in determining and advocating for appropriate statistical approaches while maintaining scientific integrity [17]. These considerations led us to a more detailed operational definition that built upon the one by Slade et al*.* [13], namely:

“Strong statistical voice” is effective advocacy for doing the right thing in important matters of statistical practice. It is achieved by clear and persuasive communication. Some elements that typically contribute to clarity include how the communication is organized, the description and application of general statistical principles, simple examples, and a list of recommended actions. Some additional elements that contribute to persuasion include collegial tone and reasonable recommendations that distinguish what must be done from what might be done.

The formal definition of strong statistical voice presented in this manuscript was developed by the authors following extensive discussions with collaborative biostatisticians and quantitative scientists around the world. An initial definition proposed by Slade et al. [13] served as an early foundation, prompting many quantitative scientists to adopt the concept and refine it within their own practice. During peer review of this manuscript, one reviewer suggested a definition centered on “having sufficient knowledge and understanding of statistics to speak confidently and effectively about how a certain set of data should be treated, because data do not speak for themselves.” The definition offered here represents a natural progression of the concept as it gains broader acceptance and moves toward becoming mainstream terminology, with the understanding that it may continue to evolve over time. Other terms that capture this sentiment, such as “strong quantitative voice”, can also be used interchangeably.

Training materials were developed to address the core pedagogic challenge of creating a scalable approach for teaching collaborative biostatisticians to engage their strong statistical voice. Strong statistical voice requires integrating technical expertise with complex interpersonal skills, as different quantitative scientists will use it in varied ways, and there is no single ‘best’ method, but failing to use it can lead to misinterpretation of findings and inefficient study design.

In collaborative research settings, a common scenario arises when a researcher requests a biostatistician to replicate an analysis from a previous study using a new dataset. While this may initially appear straightforward, a skilled biostatistician will recognize that differences in the structure, distribution, or underlying assumptions of the new dataset often necessitate a tailored analytical design. For instance, consider a clinical trial in which the original study included a relatively homogeneous patient population, while the new dataset reflects greater diversity in age, geography, and health conditions. If the biostatistician possesses a strong statistical voice, they can clearly articulate why the original design does not adequately account for the variability in the new data. By proposing a more statistically appropriate approach, they ensure that the collaboration proceeds smoothly, yielding results that are both reliable and insightful. Conversely, if the biostatistician has a ‘weak’ statistical voice, they may fail to express their concerns or convince the team to adopt a revised design, the study risks being critically flawed. From there, the best-case outcome would be for reviewers to detect these issues, leading to a rejection or a requirement to repeat the entire analysis—a costly and time-consuming setback that could have been avoided with proactive and confident communication. This example underscores the pivotal role of a strong statistical voice in fostering effective collaboration and advancing scientific integrity.

**Fig. 1:**
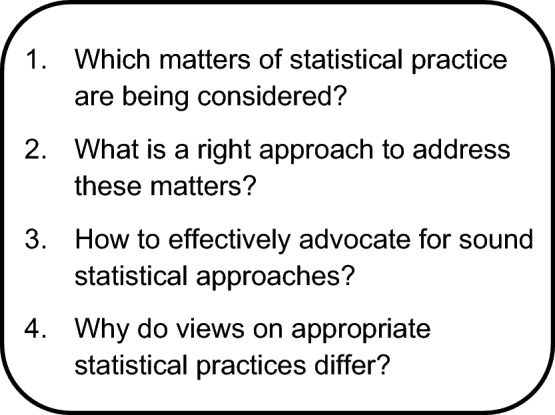
Four guiding questions for applying strong statistical voice in practice

Those who use strong statistical voice effectively are usually experienced, and it is important to separate the core skills from the role of experience. While most people recognize strong statistical voice when they see it, defining ‘what they do’ in sufficient detail to support formalized training is challenging. To support training and reflection, we identified four key questions that help clarify how strong statistical voice can be exercised in practice (Fig. 1).

## Learning Objectives

The target audience for the developed training materials is collaborative biostatisticians working in academic healthcare centers. The training materials focused on two key applications of strong statistical voice: study design discussions and manuscript review comments. These applications were selected based on systematic observations from annual staff evaluations and their central importance to biostatistical practice. Early career collaborative biostatisticians often struggle with these two applications during their first year on the job. Both applications are broadly teachable through structured approaches for applying strong statistical voice.

This approach requires biostatisticians to first identify the appropriate statistical methods and then advocate effectively for their use. To guide this, we developed the following primary learning objectives for trainees: (1) understand what is meant by strong statistical voice; (2) recognize its importance in the practice of collaborative biostatistics; (3) be able to recognize the need to apply strong statistical voice; and (4) be able to follow a structured approach to applying strong statistical voice when it is needed. Our goal in creating the educational materials was to develop a structured approach to applying strong statistical voice, which, to our knowledge, has not previously been done. To develop this training, we analyzed our experiences teaching manuscript review skills to new collaborative biostatisticians, including successful and unsuccessful approaches. From this analysis, we created a model communication framework demonstrating the effective use of strong statistical voice in manuscript review. For example, when a non-biostatistician team member drafts a manuscript, it may include unsound explanations of the statistics or an inappropriate explanation of the study design. In this case, the collaborative biostatistician on the team must point this out and suggest a more appropriate way of presenting the motivation or statistics, with the ultimate result being that the manuscript is suitably revised.

**Fig. 2:**
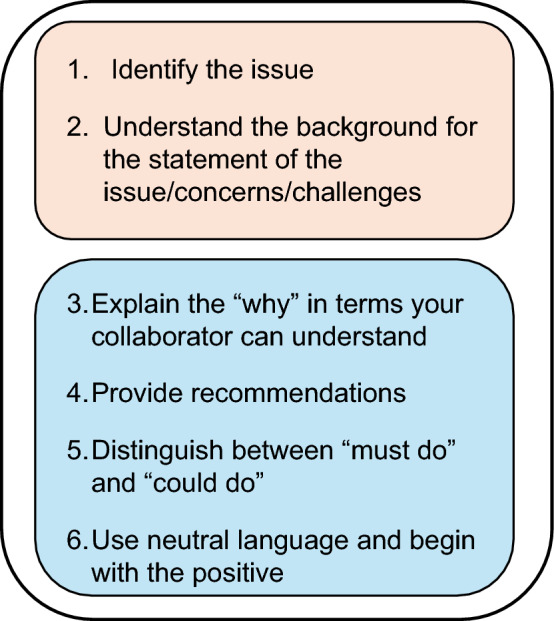
Framework for using strong statistical voice

It is important to note that the non-biostatistician colleague may have many years of research experience, while the collaborative biostatistician may have recently graduated. This dynamic can be intimidating for the collaborative biostatistician, and innovative training is needed to empower them to successfully navigate these conversations and request support from supervisors as appropriate. Breaking down these skills into clear constructs can help collaborative biostatisticians effectively communicate with their colleagues. Our framework for using strong statistical voice is outlined in six steps as illustrated in Fig. 2. Steps 1–2 of this framework should be completed before scheduling a meeting with the biomedical investigator, while Steps 3–6 guide the biostatistician’s preparation for and engagement during the discussion.

## Design of Training Materials

Sequential videos were recorded on Zoom to train collaborative biostatisticians in applying this framework. These videos served as preliminary materials for evaluation in developing a professional training program. The videos provide model communication and help collaborative biostatisticians identify characteristics contributing to effective advocacy. The video production quality was amateur, as we considered this a proof of concept. Appendix 1 provides full scripts used for each video. The primary goal was identifying the most effective content for teaching strong statistical voice. Based on the feedback and lessons learned from this evaluation (described in Sect. 5), we will develop these materials into professionally produced training videos.

We created four sequential videos introducing strong statistical voice. The videos demonstrate our framework (described above) and include a role-played conversation between a biostatistician and a scientific collaborator addressing a challenging study design issue. The videos highlight key elements, including organization, statistical principles, accessible examples, appropriate tone, action steps, and clear communication. The video series is followed by a structured exercise where trainees apply the concepts they have learned. This approach follows a “see-one, do-one” paradigm: trainees first observe applications of strong statistical voice in the videos, then practice these skills through a written exercise (Table 1).

**Table 1:** Description of the video training materials

Video number and title	Objectives	Details	Example used
1. Strong statistical voice: advocating effectively for statistical principles	Define strong statistical voice Discuss why it is important and when to use it Identify prerequisites for success	6 slides, 8 min Defining strong statistical voice Areas where statistical voice is important When to use strong statistical voice The role of effective communication and organizational knowledge (support systems) in strong statistical voice	Not applicable
2. A framework for strong statistical voice	Provide trainees a systematic approach for applying strong statistical voice	4 slides, 6 min Definition of the framework for applying strong statistical voice: Identify the issue Understand the background for the statement of the issue Explain the “why” in terms your collaborator can understand Provide recommendations Distinguish between “must do” and “could do” Use neutral language Begin with the positive Introduction to the example related to study design	Introduces an example use case for strong statistical voice related to study design
3. Identifying the issue and understanding the background	Provide a concrete example of how to identify an issue that needs the application of strong statistical voice	3 slides, 3 min Reviews the first two steps of the framework for applying strong statistical voice: Identification of the issue Understand the background for the statement of the issue	Continues the example on study design
4. Example use of strong statistical voice: a conversation with the investigator	Illustrate application of strong statistical voice in a conversation that uses the framework	Target 8–10 min A role-played conversation between biostatistician and scientific investigator that demonstrates how to put the framework for strong statistical voice into action: Explain the “why” in terms your collaborator can understand Provide recommendations Distinguish between “must do” and “could do” Use neutral language Begin with the positive	A recorded conversation between two people playing the role of scientific investigator and biostatistician Commentary on the conversation is included

The first video defines strong statistical voice, its importance, optimal application timing, and essential prerequisites for success. The second video presents a framework for applying strong statistical voice, illustrated through an example where a collaborative biostatistician must address a collaborator’s grant proposal containing a questionable study design. In this example, the scientific investigator proposed an unsound analysis because of a mistaken belief that it would increase statistical power, and the statistician’s task was to tactfully educate the scientific investigator about various statistical and study design principles and then propose a sound and acceptable way forward. Video 3 examines the example’s context and background, and Video 4 demonstrates the framework for applying strong statistical voice through a role-played interaction between a biostatistician and a scientific collaborator. Video 4 also demonstrates effective communication techniques, including “mirroring,” where the biostatistician restates the collaborator’s message to confirm mutual understanding.

An exercise in Appendix 2 complements the video series by presenting a scenario involving potential p-hacking in a manuscript analysis. The scenario features a scientific investigator’s draft manuscript containing excessive hypothesis testing. The exercise includes a model email response from a biostatistician that demonstrates the application of strong statistical voice when recommending necessary analytical modifications. Through targeted questions, learners analyze how the model email implements the strong statistical voice framework. The analysis examines key email characteristics: organization, writing clarity, explanations of statistical principles, illustrative examples, specific action recommendations, and self-contained messaging that requires no reference to previous communications. This structured approach provides more effective guidance than a simple contradiction of the scientific investigator’s methods.

In the model email, the biostatistician explains that while one statistical test maintains a 5% Type I error rate, multiple testing inflates the familywise error rate. The example also proposes specific actions to address the p-hacking concern. When guiding collaborators toward analytical changes, providing a menu of potential approaches with their respective advantages and disadvantages proves particularly effective. This also enables collaborative decision-making. The art of statistical advocacy involves being firm about essential requirements while remaining flexible on other aspects of the analysis.

This example works well if readers can follow its logic, and the biostatistician must decide if they are presenting information in an accessible and understandable manner. Not every communication needs this level of detail. Biostatisticians should tailor their communication strategy based on their collaborator’s background and working relationship. Sometimes, an in-person discussion may be more appropriate than an email like the one in the example. Another approach might be to have an executive summary precede detailed explanations like those in this email. Future iterations of this example will help the trainee think through these different scenarios.

## Evaluation of Training Materials

To evaluate our didactic training materials, we conducted a preliminary evaluation with the Duke BERD Core. This group consists of approximately 30 staff collaborative biostatisticians with master’s or doctoral degrees who work alongside biostatistical faculty and clinical research teams across the Duke School of Medicine. Their role involves participating in decisions about study design, conduct, analysis, and interpretation, necessitating regular communication and advocacy for sound statistical practice. The Duke University Health System Institutional Review Board deemed this study exempt (Pro00114556), as it focused on the efficacy of educational or instructional strategies.

This evaluation consisted of a survey aimed to identify shortcomings of the initial training material to inform future content development. A full list of questions included in the survey can be found in Appendix 3. Frequency distributions and percentages of multiple-choice questions answered on a Likert scale were summarized using SAS version 9.4 (SAS Institute Inc.) and are provided in Table 2. No adjustment of missing data was conducted as only one survey participant had incomplete responses to a single question. A summary of the free-text responses is included in the main text. The survey was anonymous, and 18 staff members participated by watching the training videos, reviewing the exercises, and completing the survey. Of the 18 participants, 12 held master’s degrees, 6 held doctorates, and their tenure at the BERD Core ranged from less than one year to 12 years, with a median of 2.75 years.

**Table 2:** Survey responses: feedback on training content for strong statistical voices

	Total (N = 18)
*This module clearly defines strong statistical voice*	
Strongly disagree	0 (0.0%)
Disagree	0 (0.0%)
Neither agree nor disagree	0 (0.0%)
Agree	11 (61.1%)
Strongly agree	7 (38.9%)
*This module provides clear examples of strong statistical voice*	
Strongly disagree	0 (0.0%)
Disagree	0 (0.0%)
Neither agree nor disagree	0 (0.0%)
Agree	8 (44.4%)
Strongly agree	10 (55.6%)
*The content in the video recordings was easy to understand*	
Missing	1 (–)
Strongly disagree	0 (0.0%)
Disagree	0 (0.0%)
Neither agree nor disagree	1 (5.9%)
Agree	6 (35.3%)
Strongly agree	10 (58.8%)
*The video recordings in this module were engaging*	
Strongly disagree	0 (0.0%)
Disagree	2 (11.1%)
Neither agree nor disagree	7 (38.9%)
Agree	7 (38.9%)
Strongly agree	2 (11.1%)
*If you are in a supervisory role: the example of a conversation around study design will assist my direct reports in improving their statistical voice*	
Not applicable	11 (61.1%)
Strongly disagree	0 (0.0%)
Disagree	0 (0.0%)
Neither agree nor disagree	0 (0.0%)
Agree	3 (16.7%)
Strongly agree	4 (22.2%)
*If you are not in a supervisory role: the example of a conversation around study design will assist me in improving my statistical voice*	
Not applicable	3 (16.7%)
Strongly disagree	0 (0.0%)
Disagree	2 (11.1%)
Neither agree nor disagree	0 (0.0%)
Agree	9 (50.0%)
Strongly agree	4 (22.2%)
*If you are in a supervisory role: the example of a written communication around p-hacking will assist my direct reports in improving their statistical voice*	
Not applicable	11 (61.1%)
Strongly disagree	0 (0.0%)
Disagree	0 (0.0%)
Neither agree nor disagree	1 (5.6%)
Agree	3 (16.7%)
Strongly agree	3 (16.7%)
*If you are not in a supervisory role: the example of a written communication around p-hacking will assist me in improving my statistical voice*	
Not applicable	3 (16.7%)
Strongly disagree	0 (0.0%)
Disagree	0 (0.0%)
Neither agree nor dsagree	2 (11.1%)
Agree	9 (50.0%)
Strongly agree	4 (22.2%)

(1) Respondents could answer questions as either ‘in a supervisory role’ or ‘not in a supervisory role’ or from both perspectives. (2) “Module” refers to the set of training videos and exercise

In addition to evaluating the training content, survey respondents were also asked about the importance of strong statistical voice and the competency of those they supervise in employing this skill. All 18 respondents strongly agreed that strong statistical voice is important to biostatistics. Six of the 18 participants responded to the question, “To what extent do you believe your direct reports are competent in using strong statistical voice?” Of these six respondents, only one said that their direct reports have advanced skills, three indicated intermediate skills, and two said their direct reports only have fundamental awareness. This emphasizes the need to develop training materials on strong statistical voice.

The survey respondents were asked about how clearly strong statistical voice was defined in the training videos and how helpful the examples were. All respondents either agreed or strongly agreed that the content in the video recordings was easy to understand, but only half agreed or strongly agreed that they were engaging. Feedback on video engagement was less favorable, likely due to the amateur production quality and insufficient focus on technical details. Future material development will focus on integrating more examples and higher quality video content.

Survey participants were also asked to provide free-text responses to give more detailed feedback about the videos and examples. Respondents were asked what they think are “the barriers to the use of strong statistical voice, e.g. Institutional barriers, global barriers”. Example responses included not identifying the issues in the first place, not knowing how to use neutral language to navigate difficult conversations, lack of training on using strong statistical voice effectively, fear of confronting scientific investigators, and power imbalances between scientific investigators and biostatisticians. Respondents also provided recommendations for improving the videos, the most common suggestions being to include more engaging examples, reduce wordiness in slides, and improve production quality. Responses span themes such as “institutional barriers” and “personal barriers”, and future work will focus on evaluating feedback using a mixed-methods approach to inform development of new training materials.

Free-text comments were also collected for the study design example and the p-hacking exercise. From these responses, we noted inconsistencies in some of the video content, which will be corrected in future development of training materials. When asked to describe “at least one component of the study design example that worked well,” respondents mentioned that it presented a realistic scenario, demonstrated successful navigation of a back-and-forth conversation, highlighted the effectiveness of asking clarifying questions as a collaborative technique, and noted that the statistician approached problem-solving by putting themselves in the scientific investigator’s position, rather than delving into the statistical details of sample size calculation. In open-text responses, participants recommended improving the study design example by maintaining consistent scientific content, providing guidance for statisticians less familiar with the subject matter, addressing investigator resistance and time constraints, and including templates for formal investigator communications.

Participants identified several effective elements of the p-hacking exercise: practical relevance, neutral tone, straightforward options for investigators, and accessible explanations without statistical jargon. Suggested improvements included adding strategies for handling investigator resistance and incorporating quantitative examples, such as false positive rates and corrected p-values, to enhance investigator understanding. Some participants noted that the email format was overly formal and lengthy, suggesting a video demonstration might better reflect typical biostatistician-investigator communication. These recommendations will inform future revisions of the materials.

## Discussion

Strong statistical voice is an important skill for successfully practicing biostatistics and related quantitative disciplines. We have described a scalable approach for teaching strong statistical voice that can be developed and used by collaborative biostatistics groups in academic healthcare centers and in other quantitative disciplines where collaboration is present. Consistent with the tenets of constructivism and active learning [18], the training methods developed progress through four stages: (1) explanation, (2) commentary on model examples, (3) practice with examples selected for pedagogic value, and (4) practice with real examples from our target audience’s experience.

In a national survey of 343 collaborative biostatisticians, ninety-two percent of the participants rated strong statistical voice as absolutely essential or important, with no one rating the skill as not important [13]. The survey findings consistently emphasized the importance of this skill, confirming its selection as a primary training focus within the Quantitative Team Science (QuanTS) Program funded by the National Institute of General Medical Sciences (NIGMS) in 2024. Preliminary evaluation results described in this manuscript have highlighted the need to improve training for quantitative scientists and guided the design of updated training materials, now more broadly referred to within the QuanTS Program as “Strong Quantitative Voice.” Because the surveyed statisticians represented only a small subset of one unit at Duke University, future evaluations will include focus groups with participants from the national community, across quantitative disciplines, to ensure broadly applicable feedback.

Our initial and generally positive evaluation of these preliminary training materials and our experience presenting similar content in formal and informal mentoring contexts suggest that strong statistical voice can be effectively taught within statistical pedagogy. Our overall interpretation of the survey responses is that the videos address an important construct; their content is reasonable but needs significant modification to make them clearer, and various details about the content of the videos and their production quality need to be improved. We found the suggestions for improvement to be both helpful and unsurprising, given the relatively early stage of development of the videos. One of the specific ideas that emerged from the free-text comments was to include “what might the statistician have done differently” as a topic for discussion. This was inspired by the various thoughtful recommendations about alternative ways of presenting the statistical content in the examples and the appreciation that there is no single “best” way of applying strong statistical voice. Additionally, there is no single example that can display strong statistical voice across different team science challenges. It will be important for future work to ensure that the framework is adaptable to a variety of contexts. Funding from NIGMS enabled development of comprehensive training materials designed to facilitate consistent application of strong statistical voice. Among these materials is a resource termed the ‘mentor guide’. This guide is designed not only to support trainees but to also empower mentors in their essential role. It includes practical exercises tailored to real-world scenarios and features specialized evaluation tools, allowing learners to assess and refine their skills actively. Through interactive guidance, the mentor guide builds a bridge between theoretical knowledge and its effective application, which is a crucial step in cultivating confidence and clarity in statistical collaboration. The full suite of resources, including professionally produced videos and mentor-led exercises, will be available at quantsprogram.com. The work of Sharp et al*.* [19] describes 10 freely accessible and professionally made videos on statistics collaborations and provides excellent training for broad communication skills. These and other relevant resources will be included as references for QuanTS participants to access for additional training.

A workgroup approach led by an experienced collaborative biostatistician offers one way to train early-stage biostatisticians in strong statistical voice. These sessions might begin with a brief lecture summarizing key concepts. Group members would then deconstruct and comment on the model communication in Appendix 1, such as identifying discussions of statistical principles related to multiple testing. Next, participants would draft written communications on new topics, for example, advocating for sample size calculations in randomized trials based on minimum clinically important differences rather than what the scientific investigators believe to be the most likely effect size. Group members would provide critical feedback on these drafts. Finally, participants would discuss examples from their statistical practice where strong statistical voice was needed, analyzing how they might have used it more effectively. The development of more curated and professional videos partnered with group mentoring sessions will provide a unique training opportunity for collaborative biostatisticians and other quantitative scientists to improve their ability to apply a strong statistical voice. The process of learning to have a strong statistical voice is also related to the ability to explain and teach foundational statistical concepts effectively. Teaching introductory statistics, particularly to non-statisticians, may also provide a unique platform to cultivate this skill.

Some of our co-authors work with and train biostatisticians outside the United States and anecdotally have observed that cultural norms may influence the biostatisticians to feel that disagreement implies some level of disrespect. We think that this can lead some biostatisticians to be more averse to using their strong statistical voice. Biostatisticians have been observed to be less inclined to identify mistakes or disagree with senior clinician-scientists even if the study design or analysis is wrong. Therefore, future work should also focus on conducting similar evaluations of statisticians outside of the United States and we should study methods to help train quantitative scientists in diverse settings. The new training iteration within the QuanTS Program will continue to use the term “Strong Quantitative Voice,” with ongoing assessments to adapt content for diverse settings. This evaluation will also aim to identify barriers to skill development and establish objective metrics for success, enabling both mentors and participants to assess progress and support professional growth.

## Appendix 1: Scripts for the Videos

### Video 1: Strong Statistical Voice: Advocating Effectively for Statistical Principles

*Title Slide:* Welcome to Strong Statistical Voice: Advocating Effectively for Statistical Principles. In this video we will define what it means to use strong statistical voice and discuss areas of practice where strong statistical voice is important for collaborative biostatisticians. We will also discuss how to identify times when you should use strong statistical voice. Subsequent videos in this course will lead you through examples.

*Slide 1:* There are likely many different ways to define strong statistical voice. Two definitions that we find relevant for collaborative biostatisticians are shown here. First, strong statistical voice can be defined as the ability to “advocate and negotiate for good and ethical statistical practices including integrating and resolving differing scientific approaches.” Second, strong statistical voice can be defined as “Effective advocacy for doing the right thing in important matters which pertain to statistical practice.” Although these definitions are slightly different, they both suggest the need for assertive but respectful advocacy for one's position to a scientific collaborator, especially when a particular application of a statistical method is not feasible or additional considerations need to be made.

*Slide 2:* There are many areas where strong statistical voice is important for collaborative biostatisticians. The following examples are common and meant to be illustrative but not exhaustive. For example, collaborative biostatisticians often find it necessary to use strong statistical voice to advocate for excellent study design, especially as it relates to alignment of the design with the research question and consideration for factors like ethical principles or safety of research participants. Many collaborative biostatisticians will encounter the need for strong statistical voice in the pre-specification of analytical plans, as well as developing data management plans that have impact on statistical issues, such as the management of missing data or outliers.

*Slide 4:* Execution of data analyses according to pre-specified plans, and focused interpretation of the results within the bounds of the study design are common areas where collaborative biostatisticians often require strong statistical voice. Finally, strong statistical voice can be helpful in the reporting of results, for example, with respect to pre-specified analytical plans or adherence to reporting guidelines that are accepted in the field or required by journal editors.

*Slide 5:* While it may seem that strong statistical voice has potential application everywhere in the research process, it is not necessarily the case that it must be applied. Instead, we argue for careful and targeted application of strong statistical voice in very specific circumstances. These circumstances are indicated by three important factors. First, the issue at hand is important. Naturally, the importance of an issue is context dependent but certainly anything that is important is worth advocating for. Second, strong statistical voice is appropriate when you have identified the right thing to do. There may be many potential “right things” that you could do, including possibly not doing anything until doing additional fact finding. Often, there is more than one potential right thing to do, with each having different pros and cons. Lastly, you should apply strong statistical voice only when you have identified a support structure within your organization and an escalation plan. For example, you may have senior staff or faculty in your management hierarchy who have particular expertise that is relevant for the important issue you are addressing. You will want to have access to these support resources prior to engaging your collaborators. Among other things, your support structure can serve as a sounding board for your planned use of strong statistical voice with your collaborators, and to provide additional expertise that underlines the importance of “doing the right thing.”

*Slide 6:* Although it is not the topic of this module, it is worth taking a moment to consider the importance of identifying your support system in the successful application of strong statistical voice. No single person, even experienced staff or faculty, are likely to know what the “right thing” to do is all the time. You may require consultation before you are confident using strong statistical voice. There are also some important issues requiring strong statistical voice that necessitate institutional support, such as from Institutional Review Boards or Conflict of Interest management committees. Especially in these cases, operating as an independent biostatistician in the absence of a support system can reduce the effectiveness of your strong statistical voice, thus increasing the risk of failure of the project.

*Slide 7:* Finally, developing your communication skills is critical to the success of strong statistical voice. One way in which communications are crucial in successful application of statistical voice is to be able to use language that your collaborators can understand. After all, you are working on the project because of expertise in biostatistics. Since others may not have your expertise it is incumbent upon you to explain technical concepts in lay language. You may find that illustrations or examples are helpful. Identifying the appropriate setting for communication, such as e-mail or in-person meetings, is also critical to the successful use of strong statistical voice. Other modules in this series address points related to communication skills and knowledge of your organizational support structure that will support your successful use of strong statistical voice.

### Video 2: A Framework for Strong Statistical Voice (with an Example Related to Study Design)

*Title Slide:* In this video we will present a framework for using strong statistical voice, and provide background details for an example application of strong statistical voice that we will use as the basis for demonstration in subsequent videos.

*Slide 2:* We recommend the following structured approach to using strong statistical voice. While the specific details of every situation that demands strong statistical voice will differ, the elements of this framework should be broadly applicable.

The initial steps to using strong statistical voice are common first steps in many technical procedures. That is, we must first identify the problem or issue that we are faced with. Second, we must understand the background that led to the issue. Equipped with this understanding we can then start to think about why the issue is concerning, keeping in mind that we must be careful to present our concerns in language that our non-statistician collaborators can understand. The reading materials for this module introduce a model for cross disciplinary communication that could be applied effectively at this stage of using strong statistical voice. The topic of effective cross-disciplinary communication is covered in more detail in other modules in this course.

Once you are able to explain to your collaborator why the issue at hand is important, you should then be able to provide recommendations for what can be done. Be careful here to distinguish between what “must” be done and what “could” be done. The difference between must and could is often context dependent and can be clearer in some cases than others. The upcoming example will help to illustrate this concept.

Finally, it is important to remain neutral. In other words, stick to the facts on the ground related to the issue at hand and avoid bringing assumptions around your collaborator’s intent or your own value judgements into your argument for what must or could be done. Finally, always remember to begin with the positive aspects of the situation rather than casting the entire issue in a negative light. This last point is quite important because after all, both you and your collaborator have a vested interest in producing high quality work. Therefore, addressing issues should be viewed constructively as a means toward the positive end that all parties are interested in achieving.

*Slide 3:* The framework for using strong statistical voice is abstract so that it can apply in a wide range of different scenarios. Now let us present a more concrete scenario in which strong statistical voice is required and illustrate how to use the framework we’ve described.

In this example, we ask you to imagine that you’re working with an investigator who has identified a funding opportunity that they would like to apply for. The application will describe a randomized, controlled clinical trial of a new intervention for smoking cessation. The field of smoking cessation has well-established standards for clinical trial design based on guidance from the Food and Drug Administration. Included among these standards are an established endpoint for assessment of efficacy. This endpoint is biochemically confirmed, self-reported smoking abstinence at 22 weeks after randomization to the new intervention or the standard care. Fifteen percent of participants randomized to the standard care are expected to have biochemically confirmed, self-reported smoking abstinence at 22 weeks. The minimum clinically important difference—in other words, the smallest increase in abstinence that might make a new intervention a compelling replacement for the standard care—is a 10% absolute increase to 25% at week 22.

*Slide 4:* Prior to meeting with you to discuss the grant proposal, the investigator has used an online calculator to determine they need 250 participants in each arm of the trial to be powered at 80% to detect the minimum clinically important difference with 2-sided 5% Type I error when the true probability of the outcome is 0.15 in the control group. The investigator has already realized that the funding opportunity is barely sufficient to support such a large trial. Armed with a basic, although incomplete, understanding of statistics the investigator wants to “get more power”.

*Slide 5:* In an attempt to “get more power” the investigator has proposed to measure the abstinence outcome repeatedly on each participant at 5, 10, 15, 20, and 22 weeks per participant. The investigator reasons that by having more observations per patient the power of the trial will be increased and they can even reduce the number of total patients required. Toward this end, the investigator has proposed that if 1 patient is worth 5 observations they can get to their target of 250 observations by reducing the total number of patients to 50 and observing each patient 5 times. The investigator proposes that this will dramatically reduce the cost of the trial, which will leave additional funds for tertiary aims of the grant, thus making a more attractive proposal to the funding organization. The investigator has given you a draft of the research plan and wants you to “fill in the statistical section of the grant.”

### Video 3: Identifying the Issue and Understanding the Background

*Title Slide:* In this video we cover the first two steps in the framework for applying strong statistical voice: identifying the issue and understanding the background.

*Slide 2:* In the example that you reviewed in the last video, there are actually many issues at play. Some of these issues are of immediate importance and others are probably less concerning, although still worthy of attention. First, you should understand that the investigator’s ultimate goal is to do a randomized, controlled trial that compares a new intervention to the standard care. The research question surrounds an outcome at 22 weeks, which is to be compared between the treatment groups. You should recognize this as essentially a cross-sectional analysis. In a desperate attempt to control the budget for the trial, the investigator has made a proposal to do a longitudinal analysis that no longer addresses the research question. Instead, the investigator’s proposal addresses the question of how the abstinence probability changes over time, which is not aligned with the FDA guidance on how to evaluate new interventions in this field. This example illustrates how limited statistical literacy can negatively impact the ability of the study to address its objectives. The example also highlights an opportunity where how strong statistical voice could be helpful.

*Slide 3:* In addition to the primary issue related to misalignment between the proposed analysis, the research question, and the regulatory guidance there are other issues that, although they might be considered of secondary importance, would be helpful to address if not only for the present study but also to assist the investigator with planning future studies. Of particular concern in this case is the investigator’s apparent lack of understanding of correlated data and how repeated measures on a single participant impact statistical power. These are difficult concepts even for statisticians to understand and would likely require some nuanced explanation, particularly since it would be necessary to use non-technical language to communicate these concepts to the investigator.

You might also have identified some other, less pressing but still important issues that are present in the investigator’s proposal. For example, powering the trial at 80% may not be adequate. Explaining why this might be the case and the impact of choosing 80% vs. 90% power, for example, are all part of statistical advocacy.

*Slide 4:* The background for the issue in this case seems clearly linked to concerns over the budget, which relates directly to the details of the funding opportunity and its suitability for the research the investigator wants to conduct. In this case, the investigator has already determined that the maximum award may not be quite enough to cover the costs of the trial and this has resulted in a backward procedure: making the design fit the budget, rather than identifying a budget to fit a design that is appropriate answer the research question. As a statistician your responsibility clearly relates to the design of the study but depending on your job role, you may not have much input on selection of the funding opportunity. However, your support system—people such as your immediate manager, their supervisor, your department chair, or other experts in the field you who collaborate with—might be well positioned to help you and the investigator identify additional funding.

### Video 4: Example Use of Strong Statistical Voice: A Conversation with the Investigator

*Statistician:* I read the details of the funding opportunity that you sent me along with your draft research plan and the FDA guidance on smoking cessation trials. I see you’ve already given this study a lot of careful thought, including some preliminary work on the sample size. It sounds like you have some concerns around the number of participants you’ll need to recruit?

*Investigator:* Yes, its going to be really important that we have as robust a design as possible for this study because I think this intervention stands a good chance of working. You know, the standard treatment for smoking cessation has been in use for a while and it’s effective but not impressively so. I think this new intervention, which combines elements of behavior modification along with pharmacotherapy, will be a game changer. The trouble is that it’s very expensive to run smoking cessation trials. There are lengthy participant interviews, carbon-monoxide breath testing, and then you’ve got the costs of producing and shipping the study drug. It all becomes overwhelming very quickly. Obviously, we need enough subjects to answer the research question but based on what I saw from an online sample size calculator the number of subjects required is not really feasible. So, I came up with this idea of increasing the amount of data we can get by measuring our outcome repeatedly on each subject. When I ran that through the online calculator the number of subjects drops from 250 per arm to 50 per arm, which is at the outer limits of what I can handle. What I’d like for you to do is to write some text about the sample size calculation for the grant proposal, which is due next week, and also to be the study statistician.

*Statistician:* Can you tell me a bit more about how you performed the sample size calculation? For example, what is your primary hypothesis?

*Investigator:* My primary hypothesis is that there will be a main effect of study group in a model with study group and time.

*Statistician:* That seems consistent with using repeated measurements for each individual to create the primary outcome variable. Which time points will you be using?

*Investigator:* The outcome variable will combine 5 weeks, 10 weeks, 15 weeks, 20 weeks, and 25 weeks.

*Statistician:* Do I correctly understand that the outcome at each time point is binary—in other words, currently a quitter or not at the 5-week observation point, currently a quitter or not at the 10-week observation point, and so forth.

*Investigator:* That’s correct.

*Statistician:* Power calculations also require some additional inputs. For example, if you were limiting yourself to the 25-week outcome only, you’d be asked to specify the proportion of quitters at 25 weeks in the intervention group and the proportion of quitters at 25 weeks in the comparison group—perhaps something like 25% and 15%. You’d also be asked to specify the desired power, and perhaps some other standard information as well. What did you specify?

*Investigator:* Interesting that you mentioned 25% and 15%—that’s what I ended up specifying.

*Statistician:* So you specified 25% and 15% at each of the time points?

*Investigator:* That’s correct.

*Statistician:* Wouldn’t the proportion of quitters drop over time?

*Investigator:* It probably would, but I wasn’t interested in that. I assumed that what really mattered to the calculation is that there was a 10% difference between the groups at each time point. So, will you be able to write the magic words to justify my sample size at 50 per arm based on the longitudinal assessments?

*Statistician:* There’s one more thing I’d like to check with you first. When I last had the pleasure of collaborating with you, I remember that you mentioned the FDA was primarily interested in long-term quitting, and that there’s a debate around whether 25-weeks is a sufficient follow-up time or, instead, whether you needed to follow patients for a full 52 weeks. Does that still hold?

*Investigator:* This isn’t a study that will be submitted to the FDA, who I was able to talk into 25 weeks if you recall, but you’re right that in my field long-term quitting is either defined by quit status at 25 or 52 weeks. My primary outcome is the 25-week measurement, and the other observations are informative but supplemental.

*Statistician:* Unfortunately, that will require us to reconsider your sample size calculation. It’s critical for the calculation to be based on the primary statistical test, which in turn tests your main study hypothesis using your primary outcome variable. Including the measures obtained prior to 25 weeks as part of the outcome combines short-term measures with a long-term measure. I think you’re stuck with an outcome which is only based on 25-weeks.

*Investigator:* Oh, I see what you mean. It’s too late to redo the budget for a larger sample size and, indeed, I don’t think a larger sample size is realistically feasible. When I originally ran the numbers for a 25-week outcome, I think my sample size of 50 per group had 80% power to detect differences like 40% in the intervention group and 15% in the comparator. Could I just use that as the assumption for the power calculation?

*Statistician:* Perhaps. Is that the minimum clinically important difference?

*Investigator:* No.

*Statistician:* I’ve always known your research to be high quality by scientific and ethical standards, so I don’t think it’s reasonable for us to pretend that 40% versus 15% is the MCID, not even to mention that the reviewers probably wouldn’t buy it. Although using a MCID to power a trial is the gold standard, I can think of a couple of alternative approaches here. Using either approach, the main and unavoidable risk is that a negative study won’t be definitive, which follows from basing your calculations on an effect size that’s larger than the MCID. One approach would be to argue that, based on the performance of other successful interventions, clinicians won’t use your intervention unless it can achieve at least a 30% improvement in outcomes. That might or might not be true—you’d know whether it is based on your knowledge of the field. The other approach would be to argue that 50 per group is the maximum sample size that’s feasible to you—maybe that allows you to keep the number of sites small and have greater quality control over the operation of the study—so you’ve kept that fixed and derived the effect size that you’re powered to detect from that. Either approach is ethical and statistically sound. If neither approach works then it might be best to not submit a proposal this time around, which would be a shame since you’ve already put in a ton of work around testing an intuitively appealing intervention. I suspect that you’ll want to give this some additional thought, but are you leaning one way or the other?

*Investigator:* Obviously, I don’t like the idea of not submitting this grant. I think your first option would probably be more persuasive to a reviewer, in other words that the intervention effect would have to be quite large to convince clinicians to change their practice. I’ll plan to do some research on this, and keep your second option in reserve as plan B.

*Statistician:* Would it be helpful to you if I drafted a short paragraph describing each option? We’d probably have to tinker with it, but perhaps putting both arguments on paper would help you decide.

*Investigator:* That would help a lot. Thanks for offering to do so.

### Video 5: Commentary on Video 4

*Commentator:* Now that you’ve had a chance to watch the video, let’s discuss how strong statistical voice was applied in this example. Recall that the first steps in applying strong statistical voice are to understand the issue and the background for the issue. In our example, the statistician was introduced to the issue in written communication between the investigator and the statistician prior to this meeting. Since this is the first time that the investigator and statistician have talked in person, the statistician takes some time to make sure their understanding of the issue and the background details is correct. The statistician does this by first stating their understanding and asking for confirmation from the investigator.

Video plays from start to 2:05.

*Commentator:* So far, the statistician has confirmed his understanding of the issue. Based on the written communications that took place before the meeting the statistician probably already has a recommendation in mind to make to the investigator. That recommendation might of course change during the conversation with the investigator if the statistician’s understanding of the issue isn’t correct going into the meeting. Naturally, this is where experience can help with smooth transitions in thinking. Luckily, in this case the statistician is able to proceed with the recommendation they had in mind prior to the meeting. However, notice that the statistician doesn’t simply give their recommendation. Instead, they begin by building an argument around their recommendation by engaging the investigator in a discussion of the study design details.

Video plays from 2:05 to 5:25.

*Commentator:* Notice that the discussion that the statistician just led was geared toward helping the investigator understand how their attempt to get “more data” actually led them astray from the original research question. Of note, this was accomplished by asking relatively simple questions that did not require explanation of technical details. For example, the statistician didn’t mention any details around correlated data or even bring up the investigator’s misunderstanding that multiple observations per patient don’t have the same impact on power as adding more patients. In fact, such technical details are tangential to the main point, which is that the design the investigator has proposed won’t answer the research question they are really interested in. Now that the statistician has “planted a seed” so to speak, he begins to make his recommendation. Again, it is a process that involves obtaining buy-in from the investigator.

Video plays from 5:25 to 6:39.

*Commentator:* Now we can see that the statistician’s argument has enabled the investigator to understand that their approach to the study design was leading them astray from their research question. The investigator now starts to look for ways he can still make the funding opportunity work for the design that is required to address the question around the 25-week outcome. Here, the statistician offers some flexibility while still guiding the investigator through a non-technical discussion around how to choose inputs for the power calculation, and ultimately leaving the choice up to the investigator while offering to help with whatever decision the investigator decides to make.

Video plays from 6:39 to 9:47.

*Commentator:* Some important things were demonstrated around using good statistical voice in this video. First, the statistician attended the meeting already informed about the issue at hand and took time at the beginning of the conversation to confirm their understanding was correct. In offering a recommendation, the statistician didn’t criticize the technical details of the investigator’s calculations because that likely would have not helped resolve the underlying issue. Importantly, the statistician focused on what must be done vs. what could be done. Namely, what must be done was to align the study design and power calculation with the study objectives. What could be done ended up to be an option for choosing how to do this, for which the statistician offered a couple possibilities. Throughout the interaction the statistician focused on the positive aspects of the problem and used neutral language.

## Appendix 2: Example of Strong Statistical Voice and an Exercise for Trainees

An investigator has circulated a draft manuscript that engages in p-hacking. The biostatistician’s comments are intended to be a self-contained communication that summarizes the problem and potentially acceptable solutions, and also that the current draft is not adequate. For the following example communication:

- Find the statement of the issue.
- Find the background for the statement of the issue.
- Find the explanation of “why” stated in terms the investigator can understand.
- Identify the recommendations that the statistician provides.
- What is the statistician saying the investigator “must do” vs. “could do”? Why do you think the statistician has prioritized certain things over others?
- Describe how neutral language is used in the communication
- Describe how the statistician begins with the positive

I read the draft manuscript with interest. Briefly, it describes a randomized trial of cord blood therapy for children with autism, and includes both safety and efficacy outcomes as described in the statistical analysis plan (SAP). The SAP treats the safety outcomes as primary and names various efficacy outcomes as secondary. One of these outcomes is the Vineland Scale (VS), which has four subscales. The SAP anticipated analyzing the VS using the overall scale and not the subscales.

The reporting of the safety outcomes appears unremarkable and sound. Most efficacy outcomes did not achieve statistical significance (nor, indeed, did they appear to exhibit a consistent signal, whether statistically significant or not). The overall VS was not statistically significant, but a measure which was composed of two of the four subscales was, and favored cord blood therapy. This “modified Vineland scale” (MVS) has not been previously used in the literature, and received considerable attention in the results section of the manuscript. In the discussion section, the MVS is proposed as the primary outcome measure in future studies. The discussion section describes a clinical rationale in support of the MVS, which is outside my area of expertise to comment on, and which I will assume is scientifically reasonable.

I believe that, by focusing on the MVS, the strength of the evidence in favor of the cord blood therapy intervention has been unintentionally overstated. I’ll explain why, and then suggest some options for how our manuscript could be revised.

*Considering multiple outcome variables has both strengths and weaknesses. A strength is that it allows for a multifaceted assessment of the impact of the intervention. A weakness is that falsely positive conclusions become more likely. Indeed, statisticians have a term for this phenomenon: the “multiple testing problem”.*

To describe the multiple testing problem, it helps to recall that every statistical test embeds the possibility of a falsely positive conclusion. For example, a typical statistical test with “alpha = 0.05” has a 5% chance of being statistically significant when the cord blood intervention is ineffective. If you perform 2 tests at alpha = 0.05, each will individually have a 5% chance of being a false positive, but the chance that at least one of the tests will be a false positive will be more than 5%. Moreover, as the number of statistical tests increases, so will the chance that at least one of these tests will be a false positive. As a result, we should approach the occasional statistically significant result with skepticism.

Some factors which increase skepticism include:

- A large number of tests
- A small number of statistically significant results
- Statistically significant results in a scientifically implausible direction
- Statistically significant results for non-primary outcome variables, especially variables which weren’t anticipated within the SAP.

In this circumstance, we observed a single result out of a large number of tests, which increases skepticism. The statistically significant result is in a scientifically plausible direction, which is encouraging. The statistically significant result was for a variable which wasn’t anticipated within the SAP, which also increases skepticism. Overall, applying these criteria, the statistical support for the conclusion that the intervention improves the MVS is, regrettably, weak. The current draft of our manuscript does not adequately address the multiple testing problem.

What might be done? Fortunately, there are a number of responses, ranging from the quantitative and formal (not recommended here) to the qualitative and informal (recommended here). As an example of a formal method, if the number of tests is small—5 for example—you might adjust the threshold for statistical significance from alpha = *0.05 to alpha* = *0.01 (i.e., 0.05 divided by the number of tests). Another formal method would be to only consider secondary outcomes such as the MVS if the primary outcome is statistically significant.*

A qualitative approach to the multiple testing problem is to describe it, and emphasize that the statistical significance of the MVS outcome should be approached with skepticism and thus requires validation. This would, I believe, meet the minimum reporting requirement.

As an additional comment, about which I won’t insist, is that a considerable space in the discussion section is devoted to the potential implications of the MVS result. Spending that much space seems more consistent with optimism about the MVS result than skepticism, but I think that at the very least the discussion should be preceded by a clear disclaimer that the MVS result has yet to be validated. Because of this, I happen to disagree with the conclusion that the MVS should definitely be the primary outcome variable in subsequent studies—perhaps we could finesse this issue for now by saying “could be considered” in the discussion section of this manuscript and worrying about the design of the next study later.

## Appendix 3: Survey Questions